# Impact of herbs and dietary supplements in patients with fibromyalgia: A protocol for a systematic review and meta-analysis of randomized controlled trials

**DOI:** 10.1097/MD.0000000000020257

**Published:** 2020-05-22

**Authors:** Juan Yang, Brent A. Bauer, Qinglong Wu, Donglin Xiong, Dietlind L. Wahner-Roedler, Tony Y. Chon, Ravindra Ganesh

**Affiliations:** a Division of General Internal Medicine, Mayo Clinic, Rochester, MN, USA; b Department of Pain Medicine, Shenzhen Nanshan People's Hospital, Shenzhen; c College of Acupuncture and Rehabilitation, Guangzhou University of Traditional Chinese Medicine, Guangzhou, China.

**Keywords:** diet, fibromyalgia, herb, randomized controlled trial, supplement, systematic review

## Abstract

**Background:**

Fibromyalgia (FM) is a common chronic pain condition that seriously affects the quality of patient lives. Its etiology, pathogenesis, and treatment still remain uncertain. Dietary supplements have been widely trialed for symptom relief for FM. The review aims to synthesize the previous literature publications to assess the impact of herbs and dietary supplements on FM patients.

**Methods:**

We will conduct a literature search in the following databases PubMed, MEDLINE, EMBASE, Cochrane Library, Scopus, and Global Health from database inception to December 2019. Clinical studies published in the English language that used human participants and address the efficacy, safety, and acceptability of herbs and dietary supplements on individuals with FM will be included. The risk of bias and quality assessment of each trial will be evaluated. If trials are enough, a meta-analysis will be conducted using software RevMan5.3, Cochrane Collaboration.

**Result:**

Our review will be the first attempt to facilitate evidence-based management using herbs and dietary supplements to treat patients with FM.

**Conclusion:**

The findings may provide a framework for future research and clinic practice in FM management.

**PROSPERO registration number:**

CRD42020149941

## 1 Introduction

Fibromyalgia (FM) is a chronic condition associated with widespread musculoskeletal pain and multiple trigger points; commonly associated with fatigue, sleep disturbances, somatic and cognitive symptoms, and often coexisting with psychiatric symptoms for at least 3 months.^[1]^ The mean worldwide prevalence of FM has been estimated to be 2.7%, ranging from values of 1.7% (Asia) to 3.1% (America).^[2]^ FM occurs more often among women as compared to men,^[3,4]^ and is the second most common diagnosis in rheumatology clinics.^[5]^ It is a pervasive and persistent disease state wherein patients often report moderate or severe pain, lower quality of life (QoF) and are often extensive utilizers of health care.^[6,7]^ The societal health care cost for an FM patient can be on the order of tens of thousands of dollars annually, which increases as disease severity increases.^[8]^ Thus, it is of crucial importance to find cost-effective therapies for FM, in order to reduce its health and economic burden. FM is a complicated condition and its etiology, pathogenesis, and treatment still remain controversial.^[9]^ Despite the use of conventional therapies (e.g., analgesics, antidepressants, calcium-channel modulators, and muscle relaxants), many FM patients do not respond satisfactorily or have limiting adverse effects (AEs) from these medications.^[10]^ This highlights the need to explore whether complementary and alternative medicine (CAM) therapies can help meet the need for symptom control.^[11]^ As one of the CAM therapies, herbs, and dietary supplements are increasingly being explored to address this unmet need.

From February to July 2003, Wahner-Roedler ^[12]^ et al conducted a survey, to assess the use of CAM therapies during the previous 6 months for the treatment of fibromyalgia in a tertiary center. Of the 289 patients responding, about 98% of patients stated using of some type of CAM, and 51% reported using at least 1 herb or dietary supplement. A consumer report indicated that almost every FM patient had used at least 1 CAM therapy for the management of FM in the past, with about 35.2% reporting having used dietary supplements.^[13]^ Such widespread use of herbs and dietary supplements highlights the need for a comprehensive review of the existing evidence in a systematic fashion.

This protocol proposes a review of the efficacy, safety, and acceptability of herbs and dietary supplements compared to control groups in patients with FM.

## 2 Methods

This protocol was registered in the international database of prospectively registered systematic reviews (PROSPERO) (CRD42020149941). https://www.crd.york.ac.uk/PROSPERO/display_record.php?RecordID=149941 on Jan 29, 2020, and complies with the Preferred Reporting Items for Systematic Reviews and Meta-Analyses guidelines.^[14]^

### 2.1 Literature search strategy

**Figure 1:**
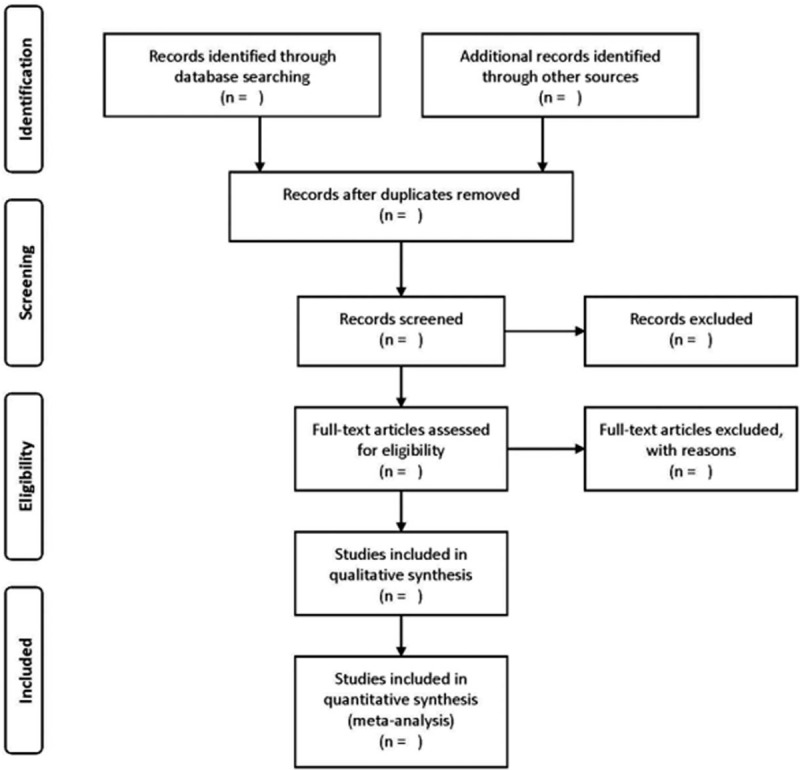
The Preferred Reporting Items for Systematic Reviews and Meta-Analyses Flow Diagram for Meta-analysis design.

A comprehensive electronic literature search will be carried on in the following databases: EMBASE, PubMed, MEDLINE, Cochrane Library, Global Health, and Scopus from database inception to December 2019. The keywords used will be “fibromyalgia” or “fibrositis” or “fibromyositis” or “FM” and “MEDICINAL plants” or “herb” or “supplement” or “vitamins”. A search will be carried out based on the list of publications available on Google Scholar. In addition, possible reviews related to FM will also be manually checked for any potentially relevant RCTs. This protocol is designed and will be performed and reported in line with the Cochrane Handbook for Systematic Reviews of Interventions^[15]^ and Preferred Reporting Items for Systematic reviews and Meta-Analyses Protocols.^[16]^ The entire process will be presented with a PRISMA flow diagram.^[16]^ (Fig. 1)

### 2.2 Study selection

The studies will be eligible if they met the following inclusion criteria.

#### 2.2.1 Participants

Subjects aged 18 or older, diagnosed with FM will be eligible for this review. No restriction on age, race, sex, socioeconomic status, or disease duration.

#### 2.2.2 Interventions

Studies evaluating the efficacy, safety and acceptability of herbs and dietary supplements on FM will be included.

#### 2.2.3 Comparators

Trials focus on the impact of herbs and dietary supplements on FM will be enrolled if they compare herbs and supplements to control groups such as no-treatment control, a placebo, or a standard treatment.

#### 2.2.4 Outcomes

The primary outcome measurements include a change in pain intensity and improvement of QoF. Pain intensity is evaluated with the Visual Analog Pain Scale Multidimensional Pain Inventory, McGill Pain Questionnaire, or the Numerical Rating Scale. QoF change is measured with the Fibromyalgia Impact Questionnaire The secondary outcome will be AE reports related to herbs and dietary supplements therapy to assess their safety and tolerability. The tertiary outcome is the dropout rate to assess the acceptability of these products to participants.

Studies will be excluded if they meet the following criteria:

(1) Duplicate studies
(2) literature review
(3) Nonrandomized controlled trials
(4) case reports
(5) studies comparing different types of herbs and dietary supplements
(6) animal experiments.

### 2.3 Data collection and management

Retrieved trials will be imported into the software reference manager EndNote X8.1. Two reviewers will independently screen the titles and abstracts of all the articles with the eligibility criteria. Retained trials will be subject to further full-text review. Data extraction will be conducted from the full-text articles of eligible studies with Excel spreadsheet including the first author, publication year, population, interventions, efficacy, follow-up, pain intensity, QoF, AEs as well as a drop-out. Two reviewers will extract the data independently. If there is any discrepancy in decisions, it will be resolved by discussion or by the involvement of a third reviewer.

### 2.4 Statistical methods

#### 2.4.1 Assessment of bias

Risk of bias of all the trials will be assessed with the Cochrane Collaboration's tool for Assessing Risk of Bias in Randomized Studies (ROBIS),^[17]^ each trial will be rated as ‘high’, ‘unclear’ or ‘low’ on the 6 domains:

(1) Selection bias
(2) Performance bias
(3) Detection bias
(4) Attrition bias
(5) Reporting bias
(6) other bias

Trials will be classified into 3 categories:

- good quality
- fair quality
- poor quality

The risk of bias will be summarized in a table.

#### 2.4.2 Study Quality assessment

The quality evidence and recommendation strength of each trial will be evaluated using the Grading of Recommendations Assessment, Development, and Evaluation tool. The assessment includes 5 items:

- risk of bias
- indirectness
- inconsistency
- imprecision
- publication bias

Each domain will be scaled as “high-, moderate-, low- or very low-quality”. Two reviewers will independently conduct the assessment. Any discrepancy between the 2 reviewers will be resolved through a consensus.

#### 2.4.3 Assessment of heterogeneity

The heterogeneity of each trial will be explored by standard Chi-square and I^2^ tests. If studies are sufficient, a subgroup analysis may be done to explore heterogeneity results.

#### 2.4.4 Assessment of reporting bias

We will draw a funnel plot and statistical test to check for the existence of reporting bias.

#### 2.4.5 Data analysis

All the collected data will be organized in an Excel spreadsheet. Study characteristics will be summarized; dichotomous outcomes will be expresses as relative risk and continuous data as mean difference with 95% confidence intervals. All the collected RCT information will be imported into RevMan5.3. If data are available, a meta-analysis will be performed. Chi-squared and I^2^ tests will be applied. If I^2^ <50%, the fixed-effect model will be used and the random-effect model will be selected if I^2^ > 50%. If a meta-analysis is not performed because of insufficient data, a qualitative synthesis of the findings from the included studies will be provided. If needed, discrepancies between the 2 reviewers will be resolved by the data provided by the original article author.

## 3 Discussion

Evidence from previous publications supports the use of herbs and dietary supplements as a possible nonpharmacological intervention for fibromyalgia. However, our understanding of the efficacy, safety, and acceptability of these products is limited. To our knowledge, this review will be the first attempt to systematically identify current clinical evidence of the role of herbs and dietary supplements in patients with fibromyalgia.

This systematic review offers a feasible means for synthesizing the evidence specific to herbs and dietary supplements on fibromyalgia, which may reveal new and novel research directions as well as advancing current management approaches to the treatment of fibromyalgia.

## 4 Strengths and weaknesses

Some possible strengths of the present work should be highlighted. Until now, to our knowledge, this systematic review is the first overview dealing with evidence of effectiveness, safety and acceptability of herbs and dietary supplements for fibromyalgia patients. To ensure that all relevant studies are included without personal or publication biases, the entire process of study selection, data extracting, and study quality assessment will be conducted by 2 reviewers independently; except for electronic database researching, Google Scholar is also performed for any additional possibly relevant RCTs.

This review also has some limitations. The literature search has language restriction (only English), and it is possible that relevant studies could be omitted from this analysis. Different types of herbs and dietary supplements may cause considerable heterogeneity in this review. Subgroup analyses may be performed to explore sources of heterogeneity based on the type of intervention.

## Acknowledgments

Dr Yang thanks the support of Sanming Project of Medicine in Shenzhen, Shenzhen Nanshan People's Hospital, Shenzhen, China for her visiting study at Mayo Clinic, Rochester MN.

## Author contributions

**Conceptualization:** Juan Yang, Ravindra Ganesh.

**Data curation:** Juan Yang, Qinglong Wu.

**Formal analysis:** Juan Yang, Donglin Xiong.

**Funding acquisition:** Ravindra Ganesh.

**Methodology:** Juan Yang, Qinglong Wu.

**Project administration:** Brent Bauer.

**Resources:** Juan Yang, Ravindra Ganesh.

**Software:** Juan Yang, Donglin Xiong.

**Supervision:** Ravindra Ganesh.

**Writing–original draft:** Juan Yang.

**Writing–review & editing:** Brent Bauer, Qinglong Wu, Donglin Xiong, Dietlind L. Wahner-Roedler, Tony Y. Chon, Ravindra Ganesh.
